# Outcome Knowledge and False Belief

**DOI:** 10.3389/fpsyg.2016.00118

**Published:** 2016-02-12

**Authors:** Siba E. Ghrear, Susan A. J. Birch, Daniel M. Bernstein

**Affiliations:** ^1^Laboratory of Knowledge, Imagination, and Development, Department of Psychology, University of British ColumbiaVancouver, BC, Canada; ^2^Laboratory of Lifespan Cognition, Department of Psychology, Kwantlen Polytechnic UniversitySurrey, BC, Canada

**Keywords:** curse of knowledge, theory of mind, hindsight bias, perspective taking, social cognition

## Abstract

Virtually every social interaction involves reasoning about the perspectives of others, or ‘theory of mind (ToM).’ Previous research suggests that it is difficult to ignore our current knowledge when reasoning about a more naïve perspective (i.e., the curse of knowledge). In this Mini Review, we discuss the implications of the curse of knowledge for certain aspects of ToM. Particularly, we examine how the curse of knowledge influences key measurements of false belief reasoning. In closing, we touch on the need to develop new measurement tools to discern the mechanisms involved in the curse of knowledge and false belief reasoning, and how they develop across the lifespan.

## Introduction

Christmas is approaching. John’s 6-year-old sister, Maggie, is very excited about Santa Claus. She has already written three letters to Santa, and is discussing the cookies she plans to leave beside the tree. As an 11-year-old, John cannot understand why his sister believes in Santa Claus. Doesn’t she realize that it is her parents who leave gifts under the tree? In this scenario John is influenced by what is called ‘the curse of knowledge’ or ‘hindsight bias’ (see Fischhoff, 1975; Camerer et al., 1989): because John knows that Santa isn’t real, it’s difficult for him to appreciate his sister’s more naïve perspective. The curse of knowledge refers to a difficulty ignoring one’s current knowledge when taking the perspective of someone less informed. This bias colors our ability to reason about the less informed thoughts of others and even recall our own previously held naïve perspectives. Considering the curse of knowledge’s profound impact on perspective taking across the lifespan, it is important to consider this bias’s role in social perspective taking, or ‘theory of mind’ (ToM).

We briefly review the literature on the curse of knowledge. We then discuss how the curse of knowledge relates to ToM, focusing on the most widely used developmental ToM measures—the classic false belief tasks. We then review literature investigating links between the curse of knowledge and ToM across the lifespan. Lastly, we suggest future research objectives that will illuminate issues critical to our understanding of the curse of knowledge and ToM.

## The Curse of Knowledge

### The Curse of Knowledge Across Development

Our brains are geared toward *acquiring* knowledge, rather than ignoring it. Although we sometimes unintentionally forget information, it is difficult to *intentionally* ‘unknow’ something (see Golding et al., 1994). Cognitive and social psychological research has investigated the pervasive nature of the curse of knowledge and its effects on social cognition and memory (see Lilienfeld et al., 2009; Roese and Vohs, 2012). Typically, researchers investigate the curse of knowledge by using either a memory design or a hypothetical design (Pohl, 2007). In a memory design, researchers ask participants to answer questions. Later, participants learn the correct answers to the questions, and must recall their original answers. Participants’ recollection of their original answers tends to be biased toward the newly learned correct answers. For example, Fischhoff and Beyth (1975) asked participants to estimate the likelihood of a set of possible outcomes of Nixon’s future visit to the USSR (e.g., ‘The USA and the USSR will agree to a joint space program’). Upon learning the outcomes of Nixon’s visit, which included a joint space flight, participants had to recall their earlier likelihood estimates of the different outcomes. Participants’ newfound knowledge of the actual outcomes to Nixon’s visit biased their recollections of their prior estimates.

In a hypothetical design, participants learn the answer to a question, and then estimate how they *would have* answered the question if they had not learned the answer, or how another individual, who had not learned the answer, would respond. For example, Fischhoff (1975) provided participants with descriptions of a historical event involving the war between the British and the Gurka. Some participants did not learn the war’s outcome, whereas others learned that ‘The British and the Gurka reached a military stalemate.’ Subsequently, participants considered several possible outcomes, including the actual outcome. For each possible outcome, participants estimated how likely it would be for a naïve peer to predict that outcome. Compared to participants who did not learn the true outcome, participants who learned the outcome estimated that naïve peers would be more likely to predict the war’s true outcome.

In curse of knowledge studies, participants’ current knowledge biases their recollections of what they previously thought and/or their ability to predict what someone else would think (Hawkins and Hastie, 1990). The curse of knowledge is robust and widespread. It occurs across a range of time intervals between exposure to the privileged outcome information and the hindsight judgment. More so, the curse of knowledge persists after explicitly warning participants about it, and providing cash incentives to avoid it (Camerer et al., 1989; Pohl and Hell, 1996). Indeed, the curse of knowledge occurs across a variety of paradigms and information types (Bryant and Brockway, 1997; Tykocinski et al., 2002; Blank et al., 2003); across cultures (Heine and Lehman, 1996; Pohl et al., 2002); and has been documented in many applied settings including business, education, and politics, as well as in academic writing and legal, governmental, and medical decision-making (e.g., Harley, 2007; Pinker, 2014). Compared to research with adults, however, the developmental literature has largely overlooked the curse of knowledge.

#### The Curse of Knowledge and Theory of Mind

We propose that there is a fundamental link between the curse of knowledge and ToM (see also, Birch and Bernstein, 2007). ToM encompasses social perspective-taking abilities that allow us to reason about our own and others’ mental states. An important aspect of ToM is the ability to infer the mental states of individuals who lack knowledge about key information and who consequently hold a false belief—a belief that is inconsistent with reality. This aspect of ToM is called false belief reasoning.

Previous research shows notable improvement in false belief reasoning between the ages of 3–5 years (for a meta-analysis, see Wellman et al., 2001). In a classic task, a child observes Sally playing with a ball and placing it in a box. Then, when Sally is away, Anne takes the ball and hides it under a basket. The child is then asked where Sally will look for the ball upon her return (e.g., Wimmer and Perner, 1983; Baron-Cohen et al., 1985). At 3 years of age, children inaccurately say that Sally will look under the basket (the ball’s current location). Rather than choosing randomly between the two locations, children are biased in the direction of their own knowledge. By 5 years of age, children tend to respond accurately, and say that Sally will look where she first hid the ball. A new wave of non-verbal false belief tasks has reported success in false belief reasoning at a much earlier age than 3 years. For instance, Onishi and Baillargeon (2005) showed that infants as young as 15 months demonstrate false belief reasoning in a violation of expectation paradigm (discussed shortly).

Why 3-year-olds fail the classic false belief task and its variants is hotly debated (Miller, 2012). One view suggests that 3-year-olds do not understand that minds can *mis*represent reality (e.g., Wellman, 1990; Perner, 1991; Gopnik, 1993). Another view suggests that developmental changes in general cognitive mechanisms (e.g., memory, language, executive functioning, and processing speed) account for the developmental change on this task. That is, 5-year-olds ability to perform the task may reflect maturation of one or more general cognitive abilities rather than a qualitative change in their conceptual understanding of the mind (Zaitchik, 1990; Fodor, 1992; Bloom and German, 2000; for other perspectives on false belief reasoning, see: Penn and Povinelli, 2007; Apperly and Butterfill, 2009; Sabbagh et al., 2013).

A variant of the latter view, the curse of knowledge account, suggests that children do not necessarily undergo a qualitative conceptual change in their understanding of the mind, but that classic false belief tasks pose the additional demand of ignoring one’s privileged knowledge—a demand that is especially problematic for young children. Consistent with this view, younger children are more susceptible to the curse of knowledge than older children and adults (Mitchell and Taylor, 1999; Birch and Bloom, 2003). The fact that most classic false belief tasks require that an outcome-knowledgeable child predict the perspective of someone less knowledgeable raises the question of how much the developmental change in the curse of knowledge bias contributes to the developmental change in false belief performance.

Previous research suggests that the curse of knowledge also affects young children’s impressions about how long they have known information. Taylor et al. (1994) found that when preschoolers learned new information (e.g., the color chartreuse) they claimed they knew it all along. They were unable to differentiate between knowledge that they learned long ago (e.g., the color red), and knowledge that they learned that day (see also Sutherland and Cimpian, 2015). Consistent with this, young children also struggle with recalling their own earlier false beliefs. For instance, in another classic false belief task, children guess the contents of a crayon box. After justifiably guessing, “crayons,” they learn it actually contains balloons. When asked to recall their earlier guess they claim they knew that there were balloons inside. They aren’t just trying to look smart—they also think that someone else will know there are balloons inside. Interestingly, young children sometimes claim that others will share their knowledge regardless of whether the others are peers, adults, or even babies (Taylor et al., 1991; e.g., Caza et al., 2016).

**FIGURE 1:**
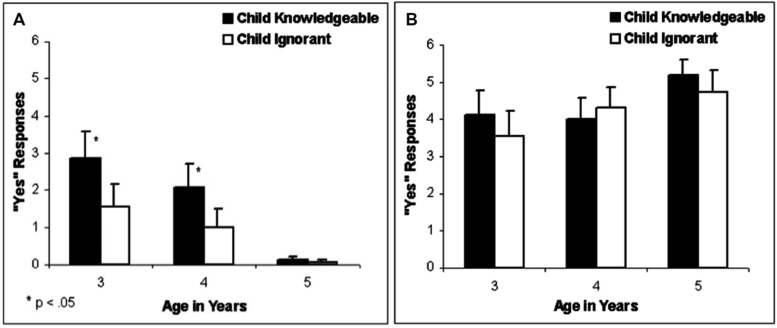
**Results from the knowledge assessment task used in Birch and Bloom (2003).** Y axis reflects the mean number of ‘yes’ responses to the question ‘Does Percy know what is inside this toy?’ across six trials. That is, the Y axis shows the mean number of times that the participants indicated that Percy would know what is inside the toy. **(A)** Shows the results for toys that Percy had not seen before. **(B)** Shows the results for toys that Percy had seen before. Black bars illustrate the performance of children who saw inside the toy (child knowledgeable), and white bars illustrate the performance of children who did not see inside the toys (child ignorant). The asterisks indicate a significant difference between the child knowledgeable and child ignorant conditions. From Birch and Bloom (2003).

The curse of knowledge is more complicated than simple egocentrism. Egocentrism predicts that an individual will overestimate how widespread his or her knowledge, or ignorance, is on a given topic. In other words, egocentrism predicts an over-attribution of one’s own perspective (whether knowledgeable or ignorant) to others. However, children only overestimate how likely other people are to share their knowledge and do not overestimate how likely other people are to share their ignorance. To show this, Birch and Bloom (2003) investigated children’s ability to infer another’s knowledge of the contents of different toys when the children either knew or didn’t know the toys’ contents. Three- to five-year-old children saw sets of toys, each containing “a special thing inside.” Children learned that Percy, a puppet, had seen what was inside one set but not the other. On half the trials, children saw what was inside both sets of toys; on remaining trials, children did not see what was inside either set, resulting in a 2 × 2 cross between Percy’s knowledge (or ignorance) and the child’s knowledge (or ignorance). When children knew the toys’ contents they overestimated Percy’s knowledge compared to when they didn’t know the toys’ contents (see **Figure 1A**). Interestingly, this bias decreased between 3 and 5 years of age, paralleling the developmental change in children’s performance on classic false belief tasks. However, when children didn’t know the toys’ contents, they didn’t overestimate Percy’s ignorance compared to when they knew the toys’ contents (see **Figure 1B**). Thus, children who didn’t see what was inside the toys were more accurate in their judgments of what Percy knew and didn’t know.

In another demonstration of the curse of knowledge’s role in children’s ToM, Lagattuta et al. (2014) tested 4- to 7-year-old children’s and adults’ ability to estimate a naïve individual’s interpretation of pictures. A cover over the pictures revealed only a small, often uninformative, portion of the picture. Some participants saw the pictures before they were covered, and others only saw the covered pictures. Participants who saw the pictures before the cover, and thus knew the pictures’ identity, overestimated the likelihood that a naïve individual would correctly guess the pictures’ identity (see also Mossler et al., 1976; Chandler and Helm, 1984; Taylor, 1988). Moreover, consistent with earlier research, children were more likely to be biased by their knowledge compared to adults (see also Epley et al., 2004; Lagattuta et al., 2010).

**FIGURE 2:**
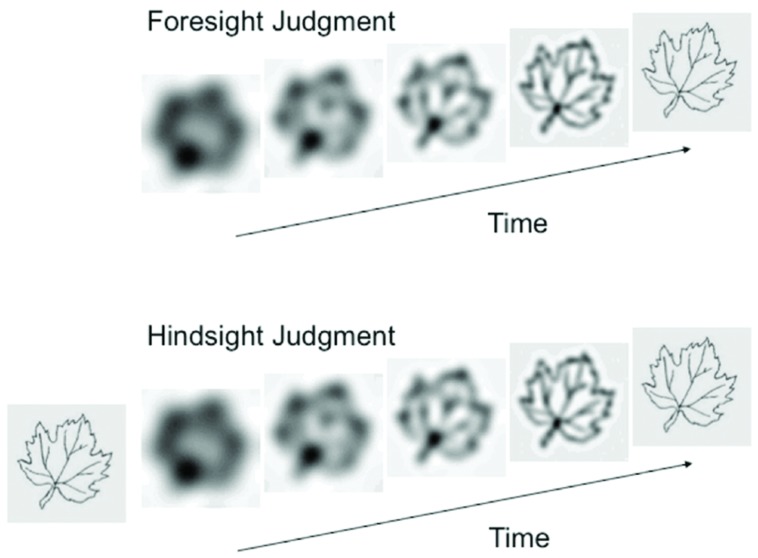
**Stimuli from the visual hindsight bias task.** In the foresight judgment condition, participants try to identify the object as it clarifies on a computer screen. In the hindsight judgment condition, participants see the object in advance of estimating when a naïve, same-age peer will identify the object. From “Hindsight bias from 3 to 95 years of age” by Bernstein et al. (2011).

Similarly, Bernstein et al. (2004) found that knowledgeable children and adults were more likely to overestimate their peers’ knowledge. In this procedure (a visual hindsight bias task, see **Figure 2** and Harley et al., 2004), 3- to 5-year-old children and adults saw degraded images of common objects that gradually clarified on a computer. In a foresight condition, participants identified an image as it clarified. In a hindsight condition, participants first saw a clear version of the image, and then estimated when a naïve peer would identify the image as it clarified. Participants who had previously seen the clear images of the objects overestimated how early their peers would be able to identify those objects. In a study examining participants from 3 to 95 years of age, Bernstein et al. (2011) found that the bias follows a u-shaped pattern across the lifespan, with preschool children and older adults exhibiting more curse of knowledge bias than older children and younger adults.

Importantly, Bernstein et al. (2007) showed a link between performance on hindsight bias tasks and classic false belief tasks among 3- to 5-year-old children. The researchers presented participants with visual hindsight bias tasks (see above) and verbal hindsight bias tasks. In the verbal tasks, children tried to answer six questions; later, children learned the answers to half the questions, and tried to recall their original answers. The researchers found that performance on both verbal and visual hindsight bias tasks correlated with performance on the classic false belief tasks. This correlation remained significant even after controlling for age, language ability, and inhibitory control.

We are not suggesting that the curse of knowledge and false belief reasoning are the same cognitive process. The curse of knowledge refers to a more general cognitive bias that applies to situations in which one needs to ignore privileged information to reason about a more naïve perspective; thus, the curse of knowledge is not limited to false belief reasoning. Moreover, false belief reasoning doesn’t *necessitate* that one have specific outcome knowledge. That is, one can deduce that an individual has a false belief about an event outcome without knowing the outcome him/herself. For example, imagine you know that Sally put her chocolates in the cupboard before leaving. After Sally leaves, her mother tells you, “Sally is not allowed to have chocolates. I’ll hide them somewhere.” Although you don’t know where her mother hid them, you can infer that Sally will have a false belief about the chocolates’ location. However, most classic false belief tasks (see Wellman et al., 2001) don’t take this approach. Rather, they make the participant knowledgeable of the specific outcome (e.g., Sally’s mother moved her chocolates to the basket), unnecessarily requiring the child to infer a false belief *and* overcome the curse of knowledge.

Consider the following study showing that adults, who understand that the mind can misrepresent reality, can be hindered by outcome knowledge when reasoning about a false belief. Birch and Bloom (2007) had adults complete a four-container version of a false belief task. In all conditions participants learned that the protagonist, Vicki, had placed her violin in the blue container before leaving. All participants then learned that the violin was moved to another container in Vicki’s absence. One group learned that it was moved to the red container; another group learned that it was moved to ‘another’ container (but did not know which container). All participants then indicated the probability that Vicki would first look in each container when she returned for her violin. Both conditions require appreciating that Vicki would hold a false belief about her violin’s location, yet adults who knew it was moved to the red container rated the probability of her acting on a false belief (i.e., first looking in the blue container) as significantly less likely. These results reveal that even adults, who undoubtedly have a conceptual understanding of false beliefs, can experience difficulty in predicting the consequences of another’s false beliefs (e.g., which action Vicki will take) when they have specific outcome knowledge (e.g., it was moved to the red container). This raises the question: how much of children’s difficulties with classic false belief tasks is due to their exaggerated curse of knowledge bias instead of a conceptual deficit in false belief reasoning.

We acknowledge that there are developments in children’s social perspective-taking abilities besides their decreasing susceptibility to the curse of knowledge. To disentangle these developmental changes, we call for new ToM tasks that reduce or eliminate the curse of knowledge and minimize other task demands. Ideally, these new tasks would employ continuous measures rather than simple pass/fail dichotomies that, by their very nature, can only produce (*seemingly*) qualitative developmental shifts (see Sommerville et al., 2013). In significant ways, the new wave of non-verbal false belief tasks reporting success at false belief reasoning in infancy (e.g., Slaughter, 2015) have made several improvements. Compared to the classic tasks, these newer tasks (a) eliminate verbal demands, (b) use more sensitive continuous measures (e.g., looking time; see Brooks and Meltzoff, 2015), and (c) require participants to make sense of someone’s actions *in retrospect*, after watching the scenario unfold (e.g., infants look longer after seeing the protagonist look for the object in a box where she should not know it is), rather than requiring participants to make *a priori predictions* (e.g., “where will Sally look for the ball?”; see Miller, 2012). This latter alteration may reduce the effects of the curse of knowledge bias that to date has only been shown to bias participants’ a priori predictions.

## Conclusion and Future Directions

The curse of knowledge is relevant to ToM. We primarily focused on the classic false belief tasks because of their widespread use, but the curse of knowledge can operate anytime someone possesses privileged information and must predict a less-informed perspective. Thus, the curse of knowledge is relevant to a wide variety of ToM tasks.

More work is needed to illuminate the mechanisms underlying the curse of knowledge and ToM. We predict that the curse of knowledge is not a result of a singular cognitive mechanism such as inhibitory control but more likely the result of two or more cognitive mechanisms and biases working in tandem (e.g., inhibitory control, working memory, and fluency misattribution) that may contribute differential effects depending on the age of the participant (e.g., see Birch and Bernstein, 2007; Groß and Bayen, 2015a,b, for discussion). The curse of knowledge has been studied extensively in young adults, with limited research in children and older adults (see Bayen et al., 2007; Coolin et al., 2015). ToM, conversely, has been studied extensively in young children but less in adults (see Birch and Bloom, 2007; Samson and Apperly, 2010; Miller, 2012; Surtees and Apperly, 2012). Future research examining these constructs across the lifespan from infancy through old age will provide a more complete understanding of the developmental changes in the curse of knowledge and ToM (and how the two relate). We believe that future research would benefit from new continuous ToM measures that can be used *across development* rather than during a small developmental window such as the infancy or preschool period (see Bernstein et al., 2011; Cassels and Birch, 2014). More continuous measures (e.g., Sommerville et al., 2013) would also aid in examining the many social and emotional correlates of individual differences in ToM and assist in identifying individuals that would benefit most from intervention techniques (see Caputi et al., 2011; Schaafsma et al., 2015). We encourage researchers to continue to move beyond false belief reasoning as the so-called ‘litmus test’ for ToM—as Paul Bloom and Tamsin German aptly noted, “there is more to ToM than false belief reasoning” (Bloom and German, 2000). Moreover, when an assessment of false belief reasoning is warranted we suggest researchers consider a variant that does not ‘curse’ the child with specific outcome knowledge, such as the aforementioned example where the antagonist hides the object in an undisclosed location or a variant akin to that used in Birch and Bloom (2007).

We, as humans, use ToM in virtually every social interaction. These abilities profoundly affect our social-emotional health, provide the foundation for moral regard and empathic concern for others, reduce prejudice and cultural intolerance, promote prosocial behavior and social competence, and predict academic achievement and better quality of life indices (for reviews see Capage and Watson, 2001; Chandler and Birch, 2010; Smith and Rose, 2011). Unfortunately, our current assessment tools limit our understanding of ToM and its correlates. The first step toward a more nuanced appreciation of ToM is to design and validate better measurement tools.

## Author Contributions

All authors listed have made substantial, direct and intellectual contribution to the work, and approved it for publication.

## Conflict of Interest Statement

The authors declare that the research was conducted in the absence of any commercial or financial relationships that could be construed as a potential conflict of interest.
